# Chemical Profile, Anti-Microbial and Anti-Inflammaging Activities of *Santolina rosmarinifolia* L. Essential Oil from Portugal

**DOI:** 10.3390/antibiotics12010179

**Published:** 2023-01-15

**Authors:** Jorge M. Alves-Silva, Maria José Gonçalves, Ana Silva, Carlos Cavaleiro, Maria Teresa Cruz, Lígia Salgueiro

**Affiliations:** 1 Institute for Clinical and Biomedical Research, Health Sciences Campus, University of Coimbra, Azinhaga de S. Comba, 3000-548 Coimbra, Portugal; 2 Faculty of Pharmacy, Health Sciences Campus, University of Coimbra, Azinhaga de S. Comba, 3000-548 Coimbra, Portugal; 3 Chemical Process Engineering and Forest Products Research Centre, Department of Chemical Engineering, Faculty of Sciences and Technology, University of Coimbra, 3030-790 Coimbra, Portugal; 4 Center for Neuroscience and Cell Biology, Faculty of Medicine, University of Coimbra, Rua Larga, 3004-504 Coimbra, Portugal

**Keywords:** anti-inflammatory, antifungal, Asteraceae, dimorphic transition, biofilm, senescence, wound healing

## Abstract

Fungal infections and the accompanying inflammatory responses are associated with great morbidity and mortality due to the frequent relapses triggered by an increased resistance to antifungal agents. Furthermore, this inflammatory state can be exacerbated during inflammaging and cellular senescence. Essential oils (EO) are receiving increasing interest in the field of drug discovery due to their lipophilic nature and complex composition, making them suitable candidates in the development of new antifungal drugs and modulators of numerous molecular targets. This work chemically characterized the EO from *Santolina rosmarinifolia* L., collected in Setúbal (Portugal), and assessed its antifungal potential by determining its minimum inhibitory (MIC) and minimum lethal (MLC) concentration in accordance with the Clinical Laboratory Standard Guidelines (CLSI) guidelines, as well as its effect on several *Candida albicans* virulence factors. The anti-inflammatory effect was unveiled using lipopolysaccharide (LPS)-stimulated macrophages by assessing several pro-inflammatory mediators. The wound healing and anti-senescence potential of the EO was also disclosed. The EO was mainly characterized by β-pinene (29.6%), borneol (16.9%), myrcene (15.4%) and limonene (5.7%). It showed a strong antifungal effect against yeasts and filamentous fungi (MIC = 0.07–0.29 mg/mL). Furthermore, it inhibited dimorphic transition (MIC/16), decreased biofilm formation with a preeminent effect after 24 h (MIC/2) and disrupted preformed biofilms in *C. albicans*. Additionally, the EO decreased nitric oxide (NO) release (IC_50_ = 0.52 mg/mL) and pro-IL-1β and inducible nitric oxide synthase (iNOS) expression in LPS-stimulated macrophages, promoted wound healing (91% vs. 81% closed wound) and reduced cellular senescence (53% vs. 73% β-galactosidase-positive cells). Overall, this study highlights the relevant pharmacological properties of *S. rosmarinifolia*, opening new avenues for its industrial exploitation.

## 1. Introduction

Despite their high mortality rates, fungal diseases are still neglected by health authorities [1]. These infections are more commonly found in the vaginal and mouth mucosae [2], with immunocompromised individuals being more susceptible [1,3]. Fungi from the *Candida*, *Aspergillus* and *Cryptococcus* genera are the principal agents that cause invasive infections [1,3]. *Candida* spp. virulence is tightly related to several factors, such dimorphic transition and biofilms [4], which are often associated with resistance to antifungal drugs [5,6]. Indeed, the concentrations needed to inhibit biofilms are much higher when compared to those for planktonic cells [6,7,8]. Dermatophytes such as those from the *Trichophyton*, *Epidermophyton* and *Microsporum* genera are often associated with superficial mycoses [9]; however, in immunocompromised hosts, they can also lead to invasive infections [10]. *Cryptococcus neoformans* are also relevant etiological agents due to their capacity to infect the central nervous system [11]. The yeast *Yarrowia lipolytica* is an opportunistic pathogen usually associated with catheter-related candidemia [12].

Fungal infections are often associated with an inflammatory response by the host due to the activation of *Toll*-like receptors 2/4 [13,14,15], which in turn activates several pro-inflammatory cascades. The nuclear factor kappa B (NF-κB) pathway is one of the most relevant pro-inflammatory pathways triggering the production several of pro-inflammatory factors, such as inducible nitric oxide synthase (iNOS) [16], an enzyme associated with nitric oxide (NO) production and often associated with chronic inflammation. In addition, the activation of the NF-κB pathway is also associated with the senescence-associated secretory phenotype (SASP) as well as with several aging phenotypes in the skin and blood [17], thus contributing to inflammaging. Accordingly, SASP is usually linked to a pro-inflammatory state, which would perpetuate the inflammatory response in the individual [18].

Furthermore, fungi are often associated with wound infection [19,20,21] and can induce wounds such as those formed during dermatophyte infections [22]. In addition, *Candida* biofilms can also colonize chronic wounds [23,24]. Therefore, wound healing is of the uttermost importance in avoiding the occurrence of systemic infections. The wound healing process requires different stages that must occur at the correct time in order to promote the resolution of the open wound [25].

Fungal infections are managed by antifungal agents; however, they are often associated with reduced activity due to several drawbacks, such as drug safety, pharmacokinetics, undesirable side effects, a narrow activity spectrum and a small number of cellular targets [26,27]. Worsening this scenario, resistant fungal strains are emerging [28,29,30,31,32] and therefore increasing the fungal infection-associated mortality rate [11]. In addition, some antifungal classes show increased toxicity [28,33], while fluconazole and other azoles only have a fungistatic effect and hence are highly predisposed to resistance [33]. Furthermore, inflammatory drugs, particularly non-steroid anti-inflammatory drugs (NSAIDs), are often associated with several adverse effects, namely nephrotoxicity, hepatotoxicity and gastric ulcers [34,35]. Thus, it is imperative to discover new anti-inflammatory and antifungal agents showing fewer side effects. 

Essential oils (EOs) are emerging as a source for new therapeutic agents due to their biological properties. Indeed, EOs appear as interesting antifungal agents due to their lipophilic nature, complex composition and capacity to strike at several targets, such as the integration of membrane structures, leading to increased permeability, the leaking of intracellular components and enzyme inactivation [36,37]. In addition, due to their highly heterogenous composition, synergic effects might be evoked, thereby promoting the biological effects of the essential oils [37], particularly as antimicrobial, anti-inflammatory, anti-senescent or wound healing agents. 

Asteraceae is a botanical aromatic family that includes around 1000 genera and over 20000 species [38] and is well known for its richness in bioactive secondary metabolites [39]. *Santolina* is a highly important genus belonging to the Asteraceae family that is widely distributed in the Mediterranean basin [40] and has several traditional uses, including as anti-inflammatory and an antimicrobial agent, among several others, ascribed to it [40]. Some species of this genus, such as *Santolina chamaecyparissus*, are traditionally used in the Mediterranean basin for the treatment of dermatitis and also as anti-inflammatory and antiseptic agents [40]. *S. semidentata*, *S. rosmarinifolia* and *S. impressa* are the sole representatives of this genus in Portugal. In Spain, *S. rosmarinifolia*’s flowerheads are traditionally used as antipyretic, anti-inflammatory, antihypertensive and hepatoprotective [40]. The same uses are also reported in Serra da Arrábida, Portugal [41]. In Algeria, this plant is used for the treatment of dermatoses [42], sometimes caused by bacteria and fungi, thus highlighting antimicrobial effects of this species. 

Having this in mind, the aim of this work was to chemically characterize the essential oil from *Santolina rosmarinifolia*, an Iberian endemism [43] collected in the Setubal region (Portugal), and to report its antimicrobial and anti-inflammatory properties, thus validating its traditional uses. In addition, and to further promote the interest in this species, other pharmacological activities were pointed out, namely its wound healing capacity and anti-senescence potential.

## 2. Results

### 2.1. Chemical Composition of the Essential Oil

Hydrodistillation produced EO with a 1.3% yield. The EO is predominantly rich in monoterpenes hydrocarbons (56.8%) and oxygen-containing monoterpenes (29.1%). Regarding individual compounds, as shown in Table 1, *S. rosmarinifolia* EO is characterized by high amounts of β-pinene (29.6%), borneol (16.9%), myrcene (15.4%) and limonene (5.7%). Sesquiterpenes were present in very low amounts, 1% for sesquiterpenes hydrocarbons and 2.7% for oxygen-containing sesquiterpenes.

### 2.2. Antifungal Activity

The antifungal activity of *S. rosmarinifolia* EO was determined against yeasts and filamentous fungi, particularly dermatophytes and *Aspergillus* spp. In addition, the effect of the EO on several relevant virulence factors of *Candida* spp. was also disclosed, with special focus on the yeast-to-hypha transition, the inhibition of biofilm formation and the disruption of preformed biofilms. 

#### 2.2.1. *S. rosmarinifolia* Inhibited the Growth of Several Pathogenic Fungi

As shown in Table 2, all tested strains were susceptible to the activity of *S. rosmarinifolia* EO. Dermatophytes were the most susceptible (Minimum inhibitory concentration, MIC = 0.07–0.14 mg/mL), followed by yeasts (MIC = 0.14–0.29 mg/mL). The EO also inhibited the growth of *Aspergillus* spp. For *C. albicans*, *C. krusei, T. mentagrophytes*, *T. rubrum* and *E. floccosum*, a fungicidal effect was observed.

#### 2.2.2. *S. rosmarinifolia* Essential Oil Inhibited Germ Tube Formation by *C. albicans*

**Figure 1:**
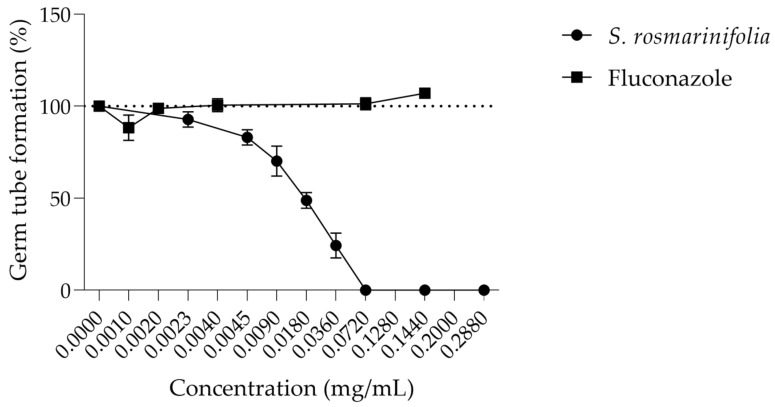
Effect of *S. rosmarinifolia* essential oil on germ tube formation of *C. albicans* ATCC 10231. Results show the mean ± SD of three independent assays made in duplicate.

Considering the relevance of dimorphic transition in *C. albicans* pathogenicity, we assessed whether the EO from *S. rosmarinifolia* could inhibit this transition. As observed in Figure 1, the EO inhibits around 50% of germ tube formation at 0.018 mg/mL (MIC/16), while fluconazole, a widely used antifungal agent in the clinic, had no effect on this virulence factor even at concentrations 200 times higher than the MIC (0.200 mg/mL). At values close to the MIC, the EO completely inhibited germ tube formation.

#### 2.2.3. *S. rosmarinifolia* Inhibited *C. albicans* Biofilm Formation

**Figure 2:**
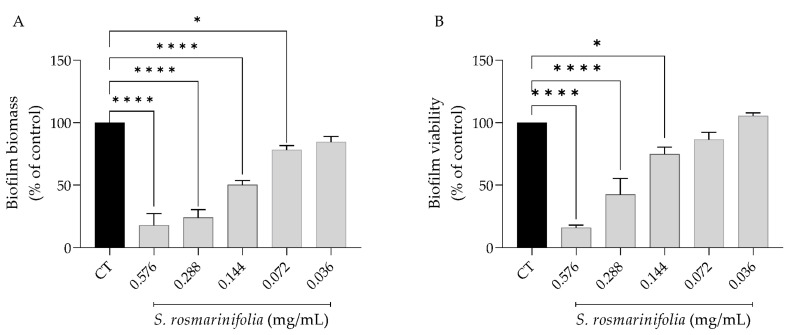
*Santolina rosmarinifolia* essential oil inhibited biofilm formation 24 h post-seeding. (**A**) Biofilm mass as measured by crystal violet assay. (**B**) Biofilm viability as assessed by XTT metabolization assay. Results show the mean ± SEM of five independent assays made in duplicate. * *p* < 0.05 and **** *p* < 0.0001 when compared to control (CT).

Fungi resistance to antifungals is often linked to the formation of biofilms [8]; therefore, inhibiting biofilm formation would prevent this resistance. Considering its promising fungicidal effect and dimorphic transition inhibition, we next assessed the effect of the EO on biofilm formation for 24 h and 48 h. As seen in Figure 2, the presence of the EO had a stronger effect on the biofilm’s mass than on its viability, since at 0.14 mg/mL (MIC/2) the EO decreased biofilm mass by approximately 50% while decreasing viability by only 25%.

**Figure 3:**
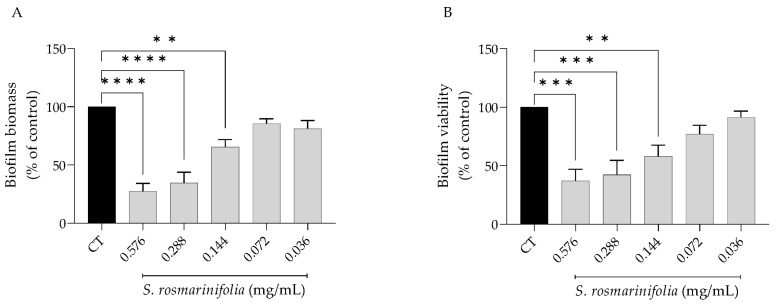
*Santolina rosmarinifolia* essential oil inhibited biofilm formation 48 h post-seeding. (**A**) Biofilm mass as measured by crystal violet assay. (**B**) Biofilm viability as assessed by XTT metabolization assay. Results show the mean ± SEM of five independent assays made in duplicate. ** *p* < 0.01, *** *p* < 0.001 and **** *p* < 0.0001 when compared to control (CT).

When the effect of the EO on biofilm formation was assessed 48 h post-seeding, it was observed that the effect was overall weaker (Figure 3). Indeed, at the same dose of 0.14 mg/mL, the EO inhibited biofilm mass by 35% and viability by 40%.

#### 2.2.4. *S. rosmarinifolia* Disrupted *C. albicans* Preformed Biofilms

**Figure 4:**
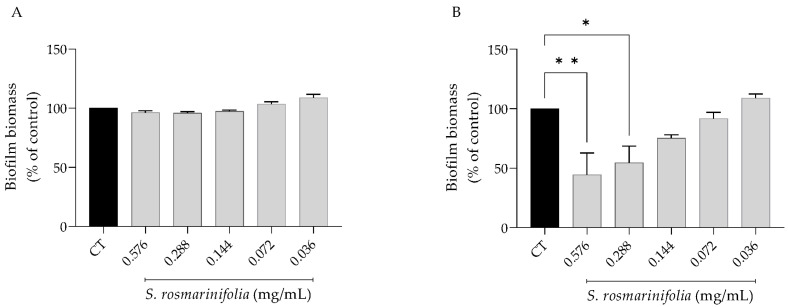
*Santolina rosmarinifolia* essential oil disrupted preformed *C. albicans* biofilms. (**A**) Biofilm mass as measured by crystal violet assay. (**B**) Biofilm viability as assessed by XTT metabolization assay. Results show the mean ± SEM of five independent assays made in duplicate. * *p* < 0.05 and ** *p* < 0.01 when compared to control (CT).

Due to their capacity to induce ecologic advantages, biofilms are difficult to eliminate, leading to refractory infections [8]; therefore, the effect of the EO on preformed *C. albicans* biofilms was assessed. As expected, the preformed biofilms were more resistant to the activity of the EO (Figure 4), as evidenced by a significant effect only on biofilm viability at 0.58 and 0.29 mg/mL (2xMIC and MIC).

### 2.3. Anti-Inflammatory Effect

Fungal infections, including those caused by dermatophytes [6,15], *C. albicans* [14,44] and *Aspergillus* [13], are often associated with an inflammatory response from the host system. Considering the relevant antifungal effect of the *S. rosmarinifolia* EO, we disclosed its anti-inflammatory potential by assessing the production of nitric oxide (NO) induced by lipopolysaccharide (LPS), a *Toll*-like receptor 4 (TLR4) agonist, in macrophages. Furthermore, the underlying mechanism of action comprised the assessment of the protein levels of inducible nitric oxide synthase (iNOS) and pro-IL1β, key pro-inflammatory mediators.

#### 2.3.1. *S. rosmarinifolia* Inhibited Nitric Oxide Production on LPS-Stimulated Macrophages

**Figure 5:**
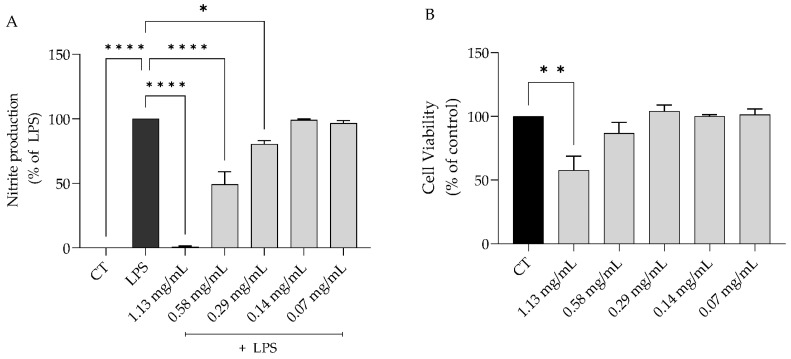
*Santolina rosmarinifolia* essential oil prevented RAW264.7 macrophage’s activation evoked by lipopolysaccharide (LPS). (**A**) Nitric oxide was quantified by measuring the amount of nitrites in the cell cultures’ supernatant using the Griess reaction, after 1h pre-treatment with the essential oil followed by 24h stimulation with LPS. (**B**) Cell viability after 24h treatment with the essential oil. Results show the mean ± SEM of three independent assays made in duplicate. * *p* < 0.05, ** *p* < 0.01 and **** *p* < 0.0001 when compared to control (CT) or LPS.

As expected, the stimulation of macrophages with LPS induced an exacerbated production of nitric oxide ([NO] = 63.5 ± 13.2 µM). Pre-treatment with the EO for 1h before the stimulation with LPS led to a dose-dependent decrease in NO production (IC_50_ = 0.52 mg/mL) (Figure 5A). To further deepen and elucidate the underlying mechanism of action, we selected the dose of 0.58 mg/mL, as we wanted to point out a dose that could present fungicidal effect and concomitantly inhibit the *C. albicans* virulence factors and, importantly, was devoid of toxicity to macrophages (Figure 5B).

#### 2.3.2. *S. rosmarinifolia* Inhibited Protein Levels of iNOS and Pro-IL1β

**Figure 6:**
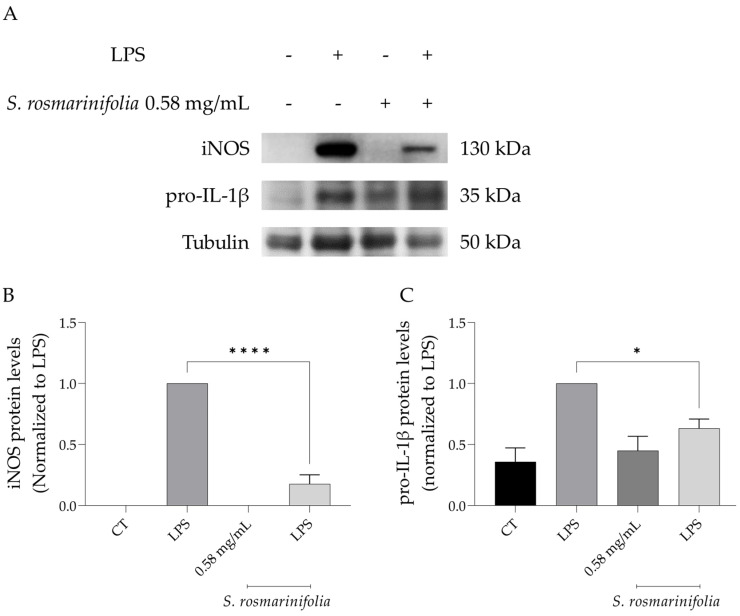
*Santolina rosmarinifolia* essential oil decreased iNOS and pro-IL-1β protein levels. (**A**) Representative western blot for iNOS and pro-IL-1β. (**B**) Relative protein levels of iNOS. (**C**) Relative protein levels of pro-IL-1β. Results show the mean ± SEM of three independent assays made in duplicate. Protein levels were normalized to tubulin and to LPS. * *p* < 0.05 and **** *p* < 0.0001 when compared to LPS (Lipopolysaccharide).

Among the signaling cascades associated with inflammation, NF-κB assumes a relevant role, and it is activated by the binding of LPS, or other ligands, to TLR4 and other pattern-recognition receptors (PPRs). The phosphorylation of NF-κB inhibitor alpha (IκB-α) by IκB kinase (IκK) leads to its proteasomal degradation, causing the release of p65 and p50 NF-κB subunits that translocate to the nucleus, triggering the transcription of several genes related to inflammation, namely *Il1b*, *Il6*, *Nos2*, *Ptgs2* and *Tnfα*. This leads to the overproduction of several pro-inflammatory cytokines, including, but not limited to, NO, resulting from the increased expression of iNOS [45,46]. As expected, stimulating the macrophages with LPS for 24h led to an increase in the protein levels of both iNOS and pro-IL-1β, the precursor form of IL-1β, the expression of which is also controlled by NF-κB [47] (Figure 6A–C). In the presence of *S. rosmarinifolia* EO, the levels of both proteins were significantly reduced, particularly for iNOS (Figure 6A–C), suggesting that the EO modulates the expression of *Nos2* and *Il1b* genes via inhibition of the NF-κB activation.

### 2.4. S. rosmarinifolia Promoted Wound Healing

**Figure 7:**
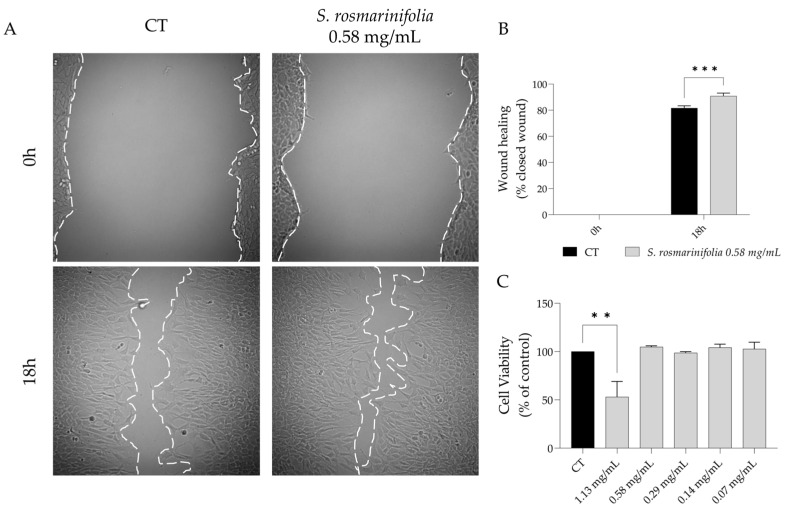
*Santolina rosmarinifolia* promoted NIH 3T3 fibroblasts migration using the scratch wound healing assay. (**A**) Representative bright-field images of NIH 3T3 fibroblasts 0 and 18h post-scratch. White line limits wound edges. (**B**) Percentage of closed wound at 0 h and 18 h post-scratch. (**C**) Cell viability of NIH 3T3 fibroblasts after 24 h of treatment with the essential oil. Results show the mean ± SEM of three independent assays made in duplicate. ** *p* < 0.01 and *** *p* < 0.001 when compared to control (CT).

*Candida* spp. are one of the most prevalent fungi found in wounds [21]. Furthermore, biofilm formation is also frequent in open wounds, which would compromise wound healing, thus perpetuating the wound and inducing chronic inflammation in the host [23,24]. Since the EO from *S. rosmarinifolia* inhibits and disrupts *Candida* spp. biofilms and decrease inflammation, we hypothesize that the EO could promote wound healing, which would decrease the risk of infection induced by biofilm formation. As seen in Figure 7, the presence of the EO significantly increased NIH 3T3 fibroblast migration in the scratch wound healing assay (91% vs. 81% of closed wound, Figure 7A) at concentrations without toxicity, thus presenting a safety profile (Figure 7B).

### 2.5. S. rosmarinifolia Decreased Etoposide-Induced Cell Senescence

It is known that inflammation leads to cell senescence [17], and that senescence itself can trigger an inflammatory response in the individual [18]. Having this in mind, we assessed whether the EO from *S. rosmarinifolia* could decrease cell senescence using etoposide as a senescence-inducing agent. 

**Figure 8:**
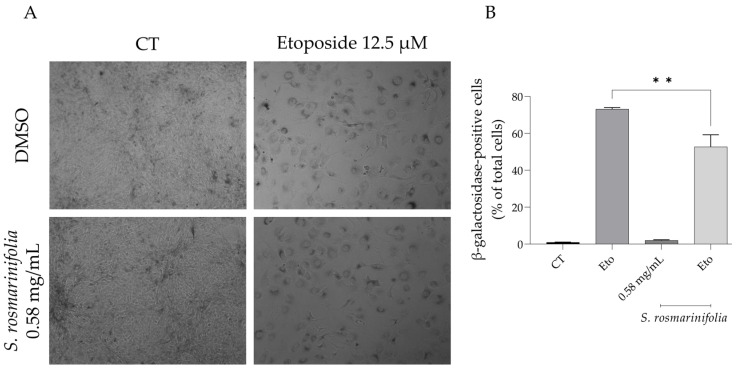
*Santolina rosmarinifolia* reduced senescence-induced β-galactosidase activity. (**A**) Representative bright-field images of NIH 3T3 cells treated with 24h of etoposide (Eto, 12.5 µM) followed by 72h in the presence or absence of *S. rosmarinifolia* (0.58 mg/mL). (**B**) Percentage of senescence-induced β-galactosidase-positive cells. Results show the mean ± SEM of three independent assays made in duplicate. ** *p* < 0.01 when compared to etoposide (Eto).

As expected, etoposide (for 24 h followed by 72 h in culture medium alone) triggered a large percentage of β-galactosidase-positive cells (Figure 8A,B); interestingly, the addition of the EO during the recovery phase significantly reduced the number of β-galactosidase-positive cells (53% vs. 73% of β-galactosidase-positive cells).

## 3. Discussion

The present study reports the chemical composition of the EO and validates the anti-inflammatory and antimicrobial properties attributed to this species. Furthermore, we report, for the first time, its capacity to inhibit *C. albicans* virulence factors, and we also disclose the molecular mechanisms underlying the reported anti-inflammatory activity. In addition, we also highlight the wound healing capacities and anti-senescence properties of the EO.

Herein, we report that the EO from *S. rosmarinifolia* collected from Portugal is mainly characterized by β-pinene, borneol, myrcene and limonene. No previous studies have been carried out on the chemical composition of the EO from Portuguese *S. rosmarinifolia.* However, some studies have evaluated the compositions of EOs from other Portuguese species, namely *S. semidentata* and *S. impressa*, which have distinct chemical profiles. *S. semidentata* presents high amounts of α-pinene, *E*-pinocarveol and pinocarvone [48], while *S. impressa* is characterized by β-pinene, 1,8-cineole, limonene, camphor and β-phellandrene [49], thus highlighting a high chemical variability among these Portuguese species. Furthermore, a recent review compared the chemical profiles of ten *Santolina* species, reinforcing the high variability among these species [40]. Regarding *S. rosmarinifolia*, it was reported that the EO from plants collected in Spain (July) has a chemical profile similar to that of the Portuguese one, namely concerning the high levels of β-pinene, limonene and myrcene. However, some differences exist, especially in the content of borneol and β-phellandrene [50]. Interestingly, a different sample from Spain, collected in April, presented high amounts of capillene, followed by β-phellandrene, myrcene, β-pinene and sabinene [51]. It is known that the season and developmental stage of the plant, as well as the genetic variability between plants, are determinant factors in the EO composition [52,53,54]. Therefore, the chemical differences between the two samples might be due to these factors. Although *S. rosmarinifolia* is an Iberian endemism [43], some studies have evaluated the composition of this species in other regions, particularly Algeria and Romania. These samples have different chemical profiles with high levels of sesquiterpenes, mainly germacrene D [55] and β-eudesmol or *ar*-curcumene [56], respectively. Another Algerian sample also showed a distinct composition, being rich in capillene [57]. The distinct chemical profiles of these samples may be due to the fact that *S. rosmarinifolia* is not indigenous to these countries. In addition, the soil composition and climatic conditions also impact chemical composition of EOs [54]. Furthermore, post-harvest treatment, storage conditions and EO extraction methods are known to affect EO composition [52,58]. Therefore, the observed differences might be attributed to any of these factors. 

Considering the emergence of fungi resistance to conventional antifungal drugs [28,29,30,31,32], we aimed to disclose the antifungal properties of *S. rosmarinifolia* essential oil. We showed that the EO was able to inhibit the growth of all tested strains, with dermatophytes being the most susceptible. Previous studies have shown that *S. rosmarinifolia* from Algeria exerts an antibacterial effect against several bacterial strains, with *Escherichia coli* and *Enterobacter aerogenes* being the most susceptible bacteria (MIC = 0.16 mg/mL) [55]. When compared with other Portuguese *Santolina* species [48,49], this species shows a more potent antifungal effect, since it was active against all tested strains. Regarding its major compounds, it was shown that β-pinene inhibited the growth of *Candida* spp., *C. neoformans*, *Trichophyton* spp. and *A. fumigatus* [59,60,61,62,63]. Several studies reported the antifungal effect of limonene against *Candida* spp., *A. fumigatus*, *T. rubrum* and several other fungi [60,64,65,66,67,68,69,70]. In contrast, borneol presented a very weak activity against several fungal strains [60,71,72,73,74]. Similarly, myrcene presents low antifungal activity [75,76,77,78,79]. The minor compounds found in the essential oil seem to play a minimal role on the antifungal activity of *S. rosmarinifolia* due to their weak antimicrobial activity. Indeed, camphor [80,81,82], 1,8-cineole [83], camphene [83,84,85,86,87,88] and α-pinene [88] are reported to exert weak inhibitory effects on the growth of several fungi. These reports suggest that the inhibitory effect of *S. rosmarinifolia* on fungal growth might be attributed predominantly to the presence of β-pinene and limonene. Considering its interesting inhibitory effects on planktonic form, we also assessed the effect of the EO of *S. rosmarinifolia* on the yeast-to-hypha transition, biofilm formation and disruption of preformed biofilms. Our results show that the EO inhibits both germ tube and biofilm formation, and, interestingly, disrupts preformed biofilms, thus reinforcing its strong antifungal effect. To the best of our knowledge, no studies have been conducted on the mechanism of action underlying the antifungal properties of *S. rosmarinifolia*. When compared with the Portuguese *S. impressa*, the EO from *S. rosmarinifolia* presents a stronger effect on germ tube inhibition [49]. Interestingly, regarding the capacity to inhibit biofilm formation, *S. rosmarinifolia* was able to affect this feature at concentrations lower than the MIC, while fluconazole is ineffective even at doses 200xMIC (data not shown). Regarding isolated compounds, a review compared the effects of different terpenes on several virulence factors and reported that β-pinene and myrcene are able to inhibit germ tube formation and biofilm development, but have, however, no effect on mature biofilms [89]. The ability of β-pinene to inhibit biofilm development was reinforced by its capacity to prevent biofilm adhesion [61]. Limonene also prevents the dimorphic transition in *C. parapsilosis* [70]. For *C. albicans*, limonene was able to inhibit germ tube formation as well as the adhesion, formation and maturation of biofilms [67]. Borneol was also able to inhibit the yeast-to-hypha transition in *C. albicans* and disrupts preformed biofilms [73]. Regarding the effect of minor compounds on *C. albicans* virulence factors, it was reported that 1,8-cineole, camphene and α-pinene were able to inhibit the dimorphic transition but had weak activity against biofilm formation and disruption [89]. Camphor is also reported to inhibit biofilm formation in both *C. albicans* and non-*albicans Candida*, as well as germ tube formation [84,90]. The results herein reported suggest that the effect of *S. rosmarinifolia* on *C. albicans* virulence factors might be due to synergic effects between the major and minor compounds of the EO.

Considering the link between fungal infections and inflammation, we assessed whether the EO from *S. rosmarinifolia* could inhibit the inflammatory response evoked by LPS in macrophages. The results demonstrated that the EO led to a dose-dependent decrease in NO production, thus validating the traditional uses ascribed to this plant. To the best knowledge of the authors, no scientific studies have been conducted yet confirming the anti-inflammatory effects of *S. rosmarinifolia*. Regarding other *Santolina* species, the anti-inflammatory potential of the EOs from *S. impressa*, *S. corsica*, *S. insularis* and *S. africana* has been previously reported. *S. africana* inhibited lipoxygenase activity with an IC_50_ of 0.065 mg/mL [91]. On other hand, *S. corsica* showed anti-inflammatory potential by decreasing the inflammatory response in bronchoalveolar lavage fluid [92]. The anti-inflammatory effects of both *S. insularis* and *S. impressa* were disclosed using the NO production evoked by LPS in macrophages, with IC_50_ of 0.55 and 0.35 mg/mL, respectively [49,93]. Regarding isolated compounds, β-pinene significantly reduced NO production in both LPS-stimulated macrophages [94] and IL-1β-activated chondrocytes [95]. Furthermore, this monoterpene is also able to inhibit the chemotaxis in neutrophils [96]. The supplementation of the mice diet with borneol significantly decreased the levels of the pro-inflammatory cytokines IL-1β and IL-6 [97]. Accordingly, several other studies reported the anti-inflammatory effect of borneol, by showing its capacity to inhibit pro-inflammatory mediators such as NO, TNF-α and IL-6 in LPS-stimulated macrophages and by decreasing fever in a model of endotoxic fever [98]. The anti-inflammatory potential of borneol was validated in animal models of mucositis [99], acne [100] and carrageenan-induced leukocyte migration [101]. Myrcene and limonene, also found in high amounts in *S. rosmarinifolia*, decreased NO production in IL-1β-activated human chondrocytes (IC_50_ = 37.3 and 85.3 µg/mL, respectively) [102] and LPS-activated macrophages, as well as in a model of pleurisy [103]. Considering the reported effects of these compounds, we hypothesize that the reduction of NO production evoked by the *S. rosmarinifolia* EO might be due to the synergistic effects between them. Having in mind the relevant activity of the EO in decreasing NO production, we further assessed its effect on the protein levels of relevant pro-inflammatory enzymes and cytokines. The results obtained demonstrated that the EO decreased the protein levels of iNOS and pro-IL-1β triggered by LPS, suggesting that *S. rosmarinifolia* might be able to inhibit the nuclear translocation of NF-κB, thus inhibiting the gene expression of pro-inflammatory mediators. Regarding the major compounds, Rufino and colleagues demonstrated that β-pinene [95] and myrcene [102] inhibited the expression of iNOS. Additionally, borneol inhibits IκBα degradation and the consequent NF-κB translocation to the nucleus [104], thus suggesting that it could inhibit the expression of pro-inflammatory mediators. Another relevant monoterpene, limonene, also decreased the protein levels of iNOS [102,105,106]. The anti-inflammatory effects reported for *S. rosmarinifolia* seem to be also attributable to the presence of minor compounds. Indeed, α-pinene inhibited NO release in IL-1β-stimulated chondrocytes [95] and LPS-stimulated macrophages [94] to a larger extent relative to the β isomer, thus suggesting that the reported activity might include a significant contribution from this minor compound. Other minor compounds, particularly 1,8-cineole [107,108,109,110,111,112], camphor [113,114,115] and camphene [116,117], are widely described as exerting anti-inflammatory effects both in vivo and in vitro. On the other hand, bornyl acetate is devoid of anti-inflammatory activity on LPS-stimulated macrophages [94].

Considering that fungal infections are often associated with chronic wounds, we hypothesized that the EO from *S. rosmarinifolia* could promote wound healing, thus decreasing the risk of wound-associated infections. Our results show, for the first time, that *S. rosmarinifolia* EO promotes wound healing, and, as far as the authors know, this is the first report on the wound healing properties of *Santolina* spp. Regarding the wound healing activity of the major compounds found in the *S. rosmarinifolia* EO, several studies have reported this activity for borneol [99,118,119] and limonene [120,121,122]. Regarding β-pinene, an essential oil from *Teucrium polium* subsp. *capitatum* rich in this compound increased wound healing [123]. Myrcene’s effect on cell migration is of a dual nature, with inhibitory effects on oral cancer cell lines [124] and stimulation of cell migration on HaCaT cells [125]. The minor compounds 1,8-cineole [112,126,127], camphor [128] and α-pinene [126] are known to promote wound healing, and thus they may also contribute to the herein reported activity of *S. rosmarinifolia*. Considering these results, we suggest that the reported activity of *S. rosmarinifolia* might be attributed to the presence of these compounds.

Since inflammation and aging/senescence are closely related, and considering the promising anti-inflammatory effects observed for *S. rosmarinifolia*, we then assessed the anti-senescence potential of the EO. The presence of the EO reduced the senescence-associated β-galactosidase activity, thus showing anti-aging effects. To the best of our knowledge, no prior studies have reported the anti-senescence effect of *Santolina* spp. Regarding isolated compounds, few studies have addressed the anti-aging effects of the major compounds found in *S. rosmarinifolia*. Myrcene improves skin aging by reducing the production of metalloproteinases and increasing the secretion of TGF-1 and type I collagen [129], and limonene increased the lifespan of the Mediterranean fruit fly and *Drosophila melanogaster* [130,131]. These results suggest that the presence of limonene and myrcene might explain the anti-senescence effect reported for *S. rosmarinifolia*. Nevertheless, the contribution of other minor compounds cannot be ruled out. Indeed, camphor, a monoterpene found in low amounts in the EO of *S. rosmarinifolia*, prevents the increased activity of senescence-associated β-galactosidase [128]. The anti-aging potential of α-pinene is also known [132]. In contrast, 1,8-cineole, another minor compound of *S. rosmarinifolia*, induced cell senescence [133]; therefore, it is likely that this compound does not contribute to and is not involved in the anti-senescence activity of *S. rosmarinifolia*.

## 4. Materials and Methods

### 4.1. Essential Oil Distillation and Analysis

#### 4.1.1. Plant Collection and Essential Oil Distillation

Aerial parts from *Santolina rosmarinifolia* were collected in July 2021, during the flowering stage, at Alto das Necessidades, Palmela, Setúbal (Portugal, 38°31′54.4″ N 8°59′19.4″ W). A voucher specimen (L. Salgueiro 87) was deposited at the Herbarium of the Faculty of Pharmacy of the University of Coimbra, Portugal. The species authenticity was validated by a taxonomist at the University of Coimbra, Doutor Jorge Paiva. The plant name was confirmed at http://www.worldfloraonline.org/taxon/wfo-0000014722 (accessed on 29 December 2022).

Prior to hydrodistillation, the plant material was air-dried for three days while protected from light. Afterwards, the material was submitted to hydrodistillation for 3 h using a *Clevenger*-type apparatus, as recommended by the European Pharmacopoeia [134] for isolation of EOs. 

#### 4.1.2. Essential Oil Analysis

The composition of the essential oil was determined by a combination of gas chromatography with flame ionization detection (GC-FID) and gas chromatography-mass spectrometry (GC-MS) as previously reported [135]. The essential oil components were identified by their retention indices on both the SPB-1 and Supelcowax-10 columns and by their mass spectra. The retention indices, calculated by linear interpolation to the retention times of C8–C23 n-alkanes, were compared with those of reference samples included in the laboratory database or reported in the literature [136]. The acquired mass spectra were compared with reference spectra from the laboratory’s database, the Wiley/National Institute of Standards and Technology (NIST) library [137] and data in the literature [138]. Relative amounts of individual components were calculated based on the GC raw data areas without FID response factor correction.

### 4.2. Antifungal Activity

#### 4.2.1. Fungal Strains

The antifungal activity of the EO of *S. rosmarinifolia* was evaluated against filamentous fungi and yeasts. The filamentous fungi included dermatophytes from clinical origin (*Epidermophyton floccosum* FF9, *Trichophyton mentagrophytes* FF7 and *Microsporum canis* FF1) and from collection (*T. mentagrophytes* var. *interdigitale* CECT 2958, *T. rubrum* CECT 2794, *T. verrucosum* CECT 2992, and *M. gypseum* CECT 2908) and *Aspergillus* spp. (*A. flavus* F44, *A. fumigatus* ATCC 46645 and *A. niger* ATCC 16404). The yeasts were represented by a *Cryptococcus neoformans* type strain (*C. neoformans* YPO186); by clinical *Candida* strains (*C. krusei* LF33, *C. guilliermondii* MAT23) and *Candida* collection strains (*C. albicans* ATCC 10231, *C. tropicalis* YPO128 and *C. parapsilopsis* ATCC 90018) and by a *Yarrowia lipolitica* type strain (*Y. lipolytica* ISA1774). 

#### 4.2.2. Macrodilution Broth Assay

The MIC and MLC of the EO were discovered using the CLSI reference protocols M27-A3 [139] and M38-A2 [140] for yeasts and filamentous fungi, respectively, as previously reported [85]. A negative control (non-inoculated medium) and a positive control (inoculated medium treated with DMSO) were included. The DMSO concentration never exceeded 1% (*v*/*v*). 

#### 4.2.3. *C. albicans* Germ Tube Formation

To assess the effect of the EO on the yeast-to-hypha transition, cell suspensions of *C. albicans* ATCC 10231 from overnight cultures on SDA were prepared in NYP medium [141], as reported [142]. Sub-inhibitory concentrations of the EO and fluconazole were used. Germ tubes were considered when the hypha was at least as long as the diameter of the blastopore. DMSO in a maximum concentration of 1% (*v*/*v*) was used as a positive control.

#### 4.2.4. *C. albicans* Biofilm Formation and Disruption of Preformed Biofilm

*C. albicans* biofilm formation was assessed according to the method described by Taweechaisupapong et al. [143] with slight modifications, as previously reported [142]. Briefly, 100 µL of a *C. albicans* suspension (1 × 10^6^ cells/mL) made in RPMI-1640 were added to 96-well pre-sterilized, polystyrene and flat-bottom microtiter plates, previously loaded with 100 µL of *S. rosmarinifolia* EO (0.036–0.576 mg/mL) serial dilutions made in RPMI. The plates were then incubated for 24h or 48h at 37 °C to allow the biofilm formation. *C. albicans* biofilm disruption assays were performed as reported [144]. Succinctly, 100 µL of *C. albicans* suspension (1 × 10^6^ cells/mL) made in RPMI was added to 96-well polystyrene microtiter plates and incubated for 24h at 37 °C. After medium removal, the wells were washed with PBS and 100 µL of serial dilutions of *S. rosmarinifolia* EO (0.036–0.576 mg/mL) made in RPMI were added and further incubated for 24 h at 37 °C. Before biofilm viability, the wells were washed three times with PBS to remove non-adherent cells. Negative (non-inoculated medium) and positive (inoculated medium treated with 1% DMSO) controls were also included. The biofilm viability was determined using the XTT/menadione metabolic assay as described [145] with some modifications, as reported [83]. The biofilm mass was quantified using the crystal violet assay as reported [89]. 

### 4.3. Anti-Inflammatory Assays

#### 4.3.1. Cell Culture

RAW 264.7 macrophages from the American Type Culture Collection (ATCC TIB-71) were cultured as previously reported [117].

#### 4.3.2. Nitric Oxide Production

The NO production was measured by quantifying the accumulation of nitrites in the culture supernatants, using the Griess reagent [146] stabilization, the macrophages were pre-treated for 1h with the EO (0.07–1.13 mg/mL) diluted in DMSO and then activated with 50 ng/mL of LPS over a period of 24h. Positive (LPS-stimulated macrophages) and negative controls (untreated macrophages) were performed. After this incubation period, equal volumes of the culture supernatants and Griess reagent [1:1 of 0.1% (*w*/*v*) N-(1-naphthyl) ethylenediaminedihydrochloride and 1% (*w*/*v*) sulphanilamide containing 5% (*w*/*v*) H_3_PO_4_] were mixed and incubated for 30 min in the dark. The absorbance at 550 nm was registered in an automated plate reader (SLT, Austria), and the nitrite concentration was determined from a sodium nitrite standard curve. DMSO at the maximum concentration used (0.4%) was already demonstrated by our group to be devoid of anti-inflammatory and cytotoxicity effects (data not shown).

#### 4.3.3. Expression of Pro-Inflammatory Proteins, iNOS and pro-IL-1β

RAW 264.7 (1 × 10^6^ cells/mL) were cultured in 6-well plates and allowed to stabilize overnight. Then, the cells were incubated with the EO (0.58 mg/mL) for 1 h, followed by LPS (50 ng/mL) activation for a period of 24 h. A negative control (untreated cells) and a positive control (LPS-only treated cells) were considered. The cell lysates were prepared as previously described [117].

A Western blot analysis was carried out to measure the protein levels of inducible nitric oxide synthase (iNOS) and pro-IL-1β. The separation, transference and membrane blocking with 5% milk made in TBS-T were performed as previously reported [147]. Afterwards, the membranes were incubated overnight at 4 °C with antibodies against iNOS (1:500; R & D Systems, MAB9502) or pro-IL-1β (1:1000; Abcam, ab9722). The membranes were then washed and incubated with secondary antibodies (1:40,000; Santa Cruz Biotechnology) conjugated with horseradish peroxidase for 1h at room temperature. After a new set of washes, the immunocomplexes were detected using a chemiluminescence scanner (Image Quant LAS 500, GE). Tubulin was used as loading control. A semi-quantitative analysis was carried out using ImageLab version 6.1.0 (Bio-Rad Laboratories Inc.).

### 4.4. Cell Migration

#### 4.4.1. Cell Culture

NIH 3T3, a mouse embryonic fibroblast cell line (ATCC CRL-1658), was cultured as previously described in our group [147]. 

#### 4.4.2. Cell Migration Assay

The cell migration was carried out using the scratch wound assay as reported [148] with slight modifications. Briefly, NIH 3T3 fibroblasts were seeded at 2.5 × 10^5^ cells/mL in 12-well plates. After 24h of growth, a scratch was made in the cell monolayer using a 20–200 µL pipette tip. The detached cells were removed by washing the cells with sterile PBS 1x. DMEM with 2% serum was added to all plates, in the presence or absence of the EO (0.58 mg/mL). Using a phase-contrast microscope, images were acquired 0 and 18 h post-scratch, and the wound area was measured using ImageJ/Fiji software. The results presented were obtained using the following equation:

(1) $$\mathrm{wound}\;\mathrm{closure}\;\left(\%\right)=\frac{A_{t=0h}-{\;A}_{t=\mathrm{xh}}}{A_{t=0h}}\times 100$$

where A_t=0h_ is the area of the wound 0h after the scratch and A_t=xh_ is the area at the different time post-scratch (0 h and 18 h).

### 4.5. Cell Viability

The study of the effects of different concentrations of the essential oil on the viability of both macrophages and fibroblasts was carried out using the resazurin reduction assay. Briefly, macrophages (0.6 × 10^6^ cells/mL) or fibroblasts (1.25 × 10^5^ cells/mL) were seeded in 48-well plates. After an overnight stabilization, different concentrations of the EO (0.07–1.13 mg/mL), diluted in DMSO, were added for 24 h. At the end of the experiment, the medium was removed and fresh medium containing resazurin (1:10) was added for 1h or 4h, for macrophages and fibroblasts, respectively. The absorbance at 570 nm with a reference filter of 620 nm was registered in an automated plate reader (SLT, Austria). Cell viability was determined using the following equation:

$$\mathrm{Cell}\;\mathrm{viability}\;\left(\%\right)=\frac{{\mathrm{Abs}}_{\mathrm{Exp}}}{{\mathrm{Abs}}_{\mathrm{CT}}}\times 100$$

where Abs_Exp_ is the absorbance (difference between 570 and 620 nm) in the different experimental conditions and Abs_CT_ is the absorbance in the control cells (no essential oil).

### 4.6. Etoposide-Induced Senescence

Senescence was assessed using the methodology previously published [149], with slight modifications. Briefly, after etoposide incubation for 24 h, cells were left to recover in the presence or absence of *S. rosmarinifolia* EO (0.58 mg/mL) for 72 h. Afterwards, cells were washed with PBS 1x, and beta-galactosidase staining was performed as described in the manufacturer’s protocol (Cell Signaling Technology). Eight images per condition were randomly acquired using a bright-field microscope. Cells showing a distinct blue color of staining were senescent. The number of positive and total cells was counted, and the percentage of senescence was calculated.

### 4.7. Statistical Analysis

Results are shown as mean values ± SEM (standard error of the mean) from a minimum of three independent experiments made in duplicate. For cell migration assays, two-way ANOVA followed by Sydák’s multiple comparison test were performed to determine statistical significance. For the remaining assays, statistical significance was determined using one-way ANOVA followed by Dunnett’s post-hoc test using GraphPad Prism version 9.5.0 (GraphPad Software, San Diego, CA, USA). Significance was considered when the *p* value was lower than 0.05.

## 5. Conclusions

This study validates the traditional uses ascribed to *S. rosmarinifolia*, particularly its antimicrobial and anti-inflammatory activities. Indeed, we report that the EO inhibits the growth of several pathogenic fungi and mitigates relevant *C. albicans* virulence factors, particularly the yeast-to-hypha transition and biofilm formation. Of relevance, we also report that the EO is able to disrupt preformed biofilms, thus strengthening the antifungal potential of this species. Regarding the anti-inflammatory activity, the EO significantly inhibits NO production as well as iNOS and pro-IL1β protein levels, probably through NF-κB pathway modulation. Interestingly, other pharmacological properties were also highlighted, specifically wound healing and anti-senescence.

Overall, this study emphasizes the industrial interest of *S. rosmarinifolia* by showing its antimicrobial and anti-inflammatory properties and concomitantly shedding light on new pharmacological activities. Furthermore, *S. rosmarinifolia* could be of interest for the management of fungal infections and the associated inflammatory response, as well as for inflammaging.

## Figures and Tables

**Table 1:** Chemical composition of the essential oil of *Santolina rosmarinifolia*.

Exp. RIa	Ref. RIa	Exp. RIa	Ref. RIb	Compound *	%
919	920	1030	1030	Tricyclene	0.2
928	930	1030	1030	α-Pinene	2.3
943	943	1075	1075	Camphene	2.6
968	970	1117	1118	β-Pinene	29.6
978	980	1160	1162	Myrcene	15.4
1011	1011	1275	1275	*p*-Cymene	0.3
1018	1019	1215	1215	1,8-Cineole	2.1
1018	1019	1204	1205	Limonene	5.7
1033	1035	1246	1245	*E*-β-Ocimene	0.3
1044	1046	1249	1249	γ-Terpinene	0.2
1074	1076	1288	1088	Terpinolene	0.2
1118	1118	1517	1515	Camphor	3.2
1119	1119	1647	1647	*E*-Pinocarveol	0.9
1144	1144	1695	1695	Borneol	16.9
1159	1158	1595	1597	Terpinene-4-ol	1.0
1166	1165	1624	1622	Myrtenal	0.9
1171	1169	1694	1692	α-terpineol	0.5
1176	1176	1786	1786	Myrtenol	0.2
1199	1196	1831	1830	*trans*-Carveol	0.0
1231	1232	1849	1852	Geraniol	0.4
1237	1235	n.d.	-	Chrysanthenyl acetate	0.2
1261	1264	1575	1574	Bornyl acetate	2.6
1362	1359	1750	1746	Geranyl acetate	0.2
1369	1368	1487	1487	α-Copaene	0.1
1442	1445	1637	1637	*allo*-Aromadendrene	0.3
1470	1466	1703	1699	Germacrene D	0.1
1477	1482	1735	1735	Bicyclogermacrene	0.1
1507	1508	1751	1751	δ-Cadinene	0.3
1522	1521	1763	1763	β-Sesquiphellandrene	0.1
1526	1525	2070	2073	Elemol	0.2
1550	1553	2113	2113	Spathulenol	0.5
1555	1557	1977	1975	Caryophyllene oxide	0.4
1574	1578	2074	2077	Guaiol	0.4
1621	1619	2180	2187	α-Muurolol	1.0
			Monoterpene hydrocarbons		56.8
			Oxygen-containing monoterpenes		29.1
			Sesquiterpene hydrocarbons		1.0
			Oxygen-containing sesquiterpenes		2.7
			Total identified		89.6

Exp. RIa: Experimental retention indices on the SPB-1 column. Ref. RIa: Reference retention indices in non-polar column. Exp. RIb: Experimental retention on the SupelcoWax-10 column. Ref. RIb: Reference retention indices in polar column. * Compounds listed according to their elution order on the SPB-1 column.

**Table 2:** Minimum inhibitory (MIC) and lethal (MLC) concentrations of *Santolina rosmarinifolia* essential oil against *Candida*, dermatophyte and *Aspergillus* strains.

Strains	*S. rosmarinifolia* Essential Oil	
	MIC ^(a)^	MLC ^(a)^
*Candida albicans* ATCC 10231	0.29	0.29
*C. tropicalis* YPO128	0.29	0.57
*C. krusei* LF33	0.29	0.29
*C. guillermondii* MAT23	0.14	0.29
*C. parapsilosis* ATCC 90018	0.29	0.57
*Cryptococcus neoformans* YPO186	0.14	0.29
*Yarrowia lipolytica* ISA 1774	0.14	0.29
*Trichophyton mentagrophytes* FF7	0.07	0.07
*T. rubrum* CECT 2794	0.14	0.14
*T. mentagrophytes var. interdigitale* CECT 2958	0.07	0.14
*T. verrucosum* CECT 2992	0.07	0.29
*Microsporum canis* FF1	0.07	0.14
*M. gypseum* CECT 2908	0.07	0.14
*Epidermophyton floccosum* FF9	0.14	0.14
*Aspergillus niger* ATCC16404	0.29	1.13
*A. fumigatus* ATCC 46645	0.14	0.57
*A. flavus* F44	0.29	0.57

^a^ MIC and MLC are expressed in mg/mL (*w*/*v*).

## Data Availability

Data will be available upon request.
